# A Modification on Hoffman's Pocket Technique With Scleral Fixation of Intraocular Lens (IOL), Case Series of Unique Scenarios

**DOI:** 10.1155/2024/7479123

**Published:** 2024-10-16

**Authors:** Ehab Alsirhy, Tariq Alanazi, Nawaf Alghamdi, Jehad Alorainy, Abdullah Alanzan, Saeed Alwadani

**Affiliations:** Department of Ophthalmology, College of Medicine, King Saud University, Riyadh, Saudi Arabia

**Keywords:** complicated cataract surgery, effective lens position, Gore-Tex sutures, Hoffman pocket, polytetrafluoroethylene sutures, SFIOL (scleral fixated intraocular lens)

## Abstract

**Purpose:** The purpose of this study is to present our experience with Hoffman's SFIOL using double-armed Gore-Tex sutures (polytetrafluoroethylene) instead of 9.0 polypropylene suture method in four cases.

**Observation:** In this case series article, we present our experience with Hoffman's SFIOL using double-armed Gore-Tex sutures (polytetrafluoroethylene) instead of 9.0 polypropylene sutures; the postoperative evaluation revealed well-centered IOL which did not exhibit any signs of instability in all four eyes, demonstrating a successful surgical outcome without observed complications.

**Conclusions and Importance:** Hoffman's SFIOL using double-armed Gore-Tex could be safe and effective methods for cases with no adequate capsular support for the IOL implantation. Compared to prolene suture, Gore-Tex gives more tensile strength and requires less manipulation during surgery minimizing postoperative inflammation which could be a valuable option in the nearby future for similar cases.

## 1. Introduction

In cases when there is no adequate capsular support for the intraocular lens (IOL) to be implanted safely in the capsular bag or the sulcus, primary or secondary IOL implantation with scleral fixation is an excellent choice for a safe implantation. It can be done by suturing and nonsuturing techniques. The latter technique is done using 9-0 polypropylene suture to fixate the IOL which is manufactured on deferent natures including foldable and PMMA rigid IOL. The choice of the IOL type is usually according to the surgeon preference; however, the implantation using foldable IOL is a good choice for that procedure since it provides better maneuverability and smaller wound size as compared to the PMMA IOL (rigid polymethyl methacrylate IOL), which usually needs 6 ± 0.5 − mm incision size [1, 2], thus adding more advantages, including globe stability and preventing hypotony and its related complications. However, PMMA IOL (which will need larger corneal wound) still a valid option. One of the well-described surgical techniques is known as “Hoffman pocket” which is introduced by Hoffman and his colleagues, first time in 2006 in which an intrascleral pocket (tunnel) is created down the hill from the cornea posteriorly which is meant to provide a good coverage of the knots without the need for conjunctival peritomy. These scleral pockets should be located 180 degrees away from each other horizontally (nasally and temporally). Moreover, Hoffman's technique is superior to other techniques by sparing the conjunctival tissues (as there is no need for periotomy), with less time consuming and less scar formation [1].

Originally, the type of the used suture is 9-0 polypropylene suture to fixate the IOL on the sclera.

In this case series, we will present our experience in dealing with unique examples of aphakia, complicated cataract surgery and postcataract surgery IOL opacification by doing a modified Hoffman pocket IOL (foldable, hydrophobic type) scleral fixation with Gore-Tex sutures instead of polypropylene sutures which is a modification on the original Hoffman's SFIOL technique.

## 2. Case Description

### 2.1. Case 1

**Figure 1:**
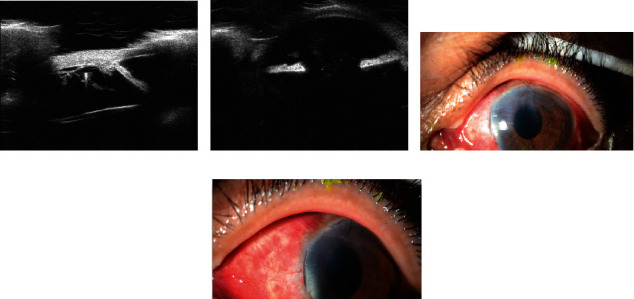
(a–d) Representing the patient's preoperative and postoperative eye findings including anterior segment UBM and slit lamp photos of Case Number 1.

A 64-year-old gentleman underwent phacoemulsification and posterior capsular implantation of the IOL with capsular tension ring implantation under local anesthesia in the left eye, which was complicated by severe zonular weakness probably due to pseudoexfoliation. His vision was stable postoperatively for a relatively short time. Then, over the follow-up visits in the clinic, we found that he developed an inferior subluxation of the IOL in the left eye. His vision was 20/100–20/25, respectively, using Snellen's chart with bilaterally normal intraocular pressure. Then, we counseled and obtained consent for IOL implantation, followed by scleral fixation using Gore-Tex sutures. The surgery was done using Hoffman's SFIOL technique without complications. His postoperative examination revealed 20/25 visual acuity by Snellen's chart bilaterally. Intraocular pressure was WNL, showing a clear cornea with a deep and quiet anterior chamber with normal iris and pupil as well as well-centered IOL. The photos were taken preoperatively showing anterior segment UBM presenting the subluxation of the IOL. The slit lamp photos were taken postoperatively, showing the relatively clear cornea with suture securing the main wound with the temporal and nasal pocket edges of the Hoffman's surgery with the fixating sutures being buried (Figures 1(a), 1(b), 1(c), and 1(d)).

### 2.2. Case 2

**Figure 2:**
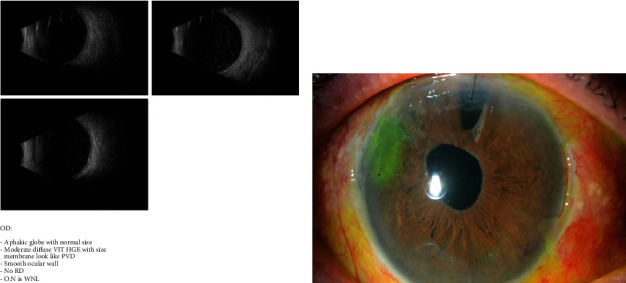
(a, b) Showing the B scan preoperatively and then the slit lamp photos postoperatively of Case Number 2.

A 68-year-old gentleman who is known to have pseudoexfoliation in his right eye had subjected to cataract surgery 6 months prior to presentation which was complicated by nucleus drop and followed by posterior vitrectomy, and his eye left aphakic with no potential sulcus support had referred to our center for secondary IOL fixation. We accordingly counseled the patient for doing the right eye scleral fixated IOL using Hoffman's technique assisted by Gore-Tex sutures under local anesthesia. His preoperative examination revealed counting fingers vision at 20/60 according to Snellen's chart and normal intraocular pressure. Globe aphakia was found in his right eye with a flat retina. The surgery was done successfully. His postoperative examination showed visual acuity of HM (with a good potential for better visual acuity since this visual acuity was taken on Day 1 postoperatively and the patient did not attend the next follow-ups based on the optic disc normality and flat and normal retina pre- and postoperatively). Preoperative B scan was taken for the patient, showing the bellow. The next photo shows the postoperative result with a relative corneal clarity with the suture securing the main superior wound; the fixating sutures on the nasal and temporal sides were buried under the scleral pockets. The IOL was stable in its place and well-centered. The superior iridectomy was done previously outside our hospital. The corneal epithelial defect seen in the photo was treated until it healed (Figures 2(a) and 2(b)).

### 2.3. Case 3

**Figure 3:**
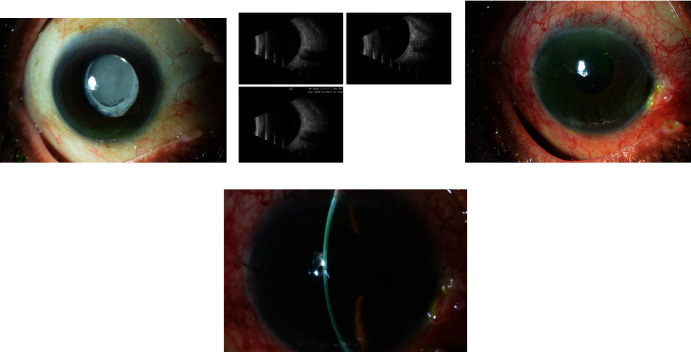
(a–d) Showing the pre-operative slit lamp photo and B scan and post-operative slit lamp photos of Case Number 3.

A 78-year-old male patient did his cataract surgery ten years back before his presentation to our ophthalmology clinic complaining of a decreased vision in the operated eye (the right eye). His vision was hand motion due to an IOL opacification and crystallization (the view to the posterior pole). B scan was done to the patient and showed unremarkable result. The decision was to counsel the patient for doing IOL extraction and exchange ± scleral fixation of the IOL using Hoffman's pocket technique, and he agreed on that. Surgery was done to the patient, his vision was hand motion, and the IOL was stable in place (Figures 3(a), 3(b), 3(c), and 3(d)).

### 2.4. Case 4

**Figure 4:**
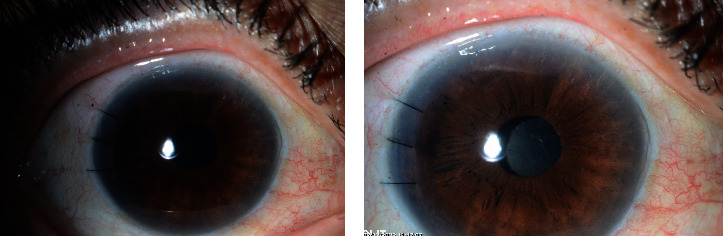
(a, b) Showing the postoperative photos of Case Number 4.

She was a 50-year-old female patient with a medical history of hypertension and diabetes mellitus. She came to the clinic for poor vision since a long time ago (≥ 10 years back). Her visual acuity was CF 4 ft in the right eye, 20/200 to 20/60 ph in the left eye by Snellen chart. Her intraocular pressure was 15/12 mmHg by GAT in both eyes, respectively. The cornea was clear bilaterally with deep and quite anterior chambers. The iris was within normal limits bilaterally. The degree of cataract was nuclear sclerosis +++ (NS+++) in both eyes. The view to the posterior pole was unclear, mandating the need for a B scan which showed a phakic globe bilaterally with a flat retina and healthy optic discs. The patient was sent to the preoperative ancillary workup, including endothelial cell count, which showed normal values to go with phacoemulsification and IOL implantation in the bag. Intraoperatively, the case was challenging due to a small capsulorhexis since it was intended to implant a toric IOL in the bag. However, a dialysis of the zonules happened due to multiple trials to extract the hard lens from the bag. It was > 180 degrees. Then intraoperatively, we converted the surgery to anterior vitrectomy guided by triamcinolone injection with scleral fixated IOL implantation using Hoffman's technique with Gore-Tex double-armed suture nasally. The temporal part of the IOL was supported by the capsular bag since there was a good and stable amount of bag and zonules temporally. The next day, a postoperative examination revealed her vision was 20/400 in the right eye (the operated eye). The eye is quite with 2+ corneal edema with a deep and quiet anterior chamber. The vision then reassessed after 3 weeks postoperatively, and it was 20/30 in the operated eye. The IOL was stable without any abnormal movement, supported by the remaining bag tissue and the nasal scleral sutures buried under the scleral pocket (Figures 4(a) and 4(b)).

## 3. Surgical Technique of SFIOL Using Hoffman Pocket and Gore-Tex

**Figure 5:**
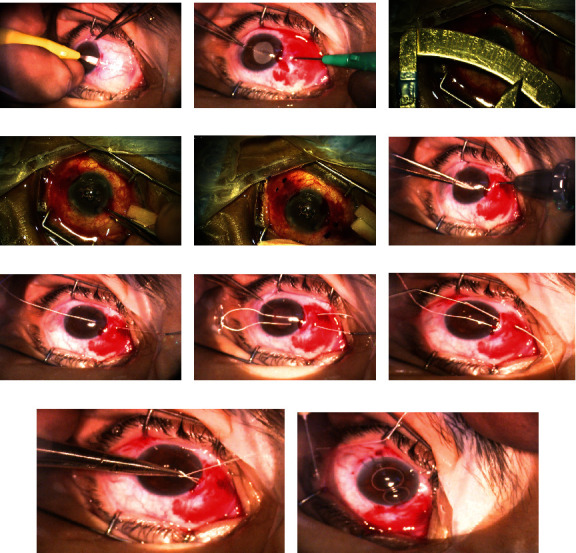
(a–k) Representing our modification on Hoffman's pocket technique for IOL fixation on the sclera (refer to the Surgical Technique of SFIOL Using Hoffman Pocket and Gore-Tex section for more descriptive details).

In a planned procedure for secondary implantation of a scleral fixated IOL, preferably before any opening to the eye (the eye still rigid), the two opposed Hoffman pockets are constructed in both sides temporally and nasally (3 and 9 o'clock) with little elevation (about 20 degrees), a mark done. The idea of Hoffman pocket is to create an intrascleral tunnel backward (or down the hell as mentioned by Dr. Hoffman), starting by creating a half-thickness perilimbal corneal incision (arch-shape) with a width of about 2–3 clock hours on each side; the tunnel formation is contemplated by crescent knife (preferably 2.25 mm, angled tip, bevelled up); and the thickness of the tunnel is guided by detecting the blade through the semitransparent conjunctival-scleral tissues and aided by pulling the globe by a toothed forceps to the opposite site (e.g., pulling the globe nasally when fashioning the temporal pocket and vice versa) (Figure 5(a)). Some resultant bleeding while forming the pockets is just to be omitted, as it stops spontaneously, but need to pay attention that blood should not leak inside the globe. Some cases needed triamcinolone-guided anterior vitrectomy to be sure there is no vitreous bands in the anterior chamber. The next step is to make two openings in the scleral wall on each side (temporal and nasal) with 2-mm distance between them and 1.5–2.0 mm back to the limbus over the roof (conjunctival side) of the previously constructed tunnels using MVR knife size 21G; however, in case 3, in this series, we decided to go more posterior at pars plana (3.5–4.0 mm) with the same manner, the 3.5 for the superior arm of the loop and the 4.0 for the inferior one (Figures 5(b), 5(c), 5(d), and 5(e)). Then, the lens is extracted from the injecting system for manual implantation. We used the foldable IOL RayOne (Rayner company, UK) the hydrophobic type which comes with a dual haptic (loop-shaped), and quadrangle optic design, although it comes preloaded, but we remove it out to be able to thread the suture in the looped haptics. Then, the main incision is created by 2.5-mm keratome on superior location (away from the pockets) for introducing the lens. The Gore-Tex suture comes with bulky needle (which is specifically designed for the heart surgery not for the eye surgery), so we cut it. The suture itself (CV 8, which is roughly equal to 7-0 on the USP scale) is hydrophobic, monofilament, and nonabsorbable and has a high tensile power. Its shape is not cylindrical, but rather has a flat configuration; all these characteristics facilitate the handling to a great degree. Then, by using an intraocular microforceps (with straight serrated tips forceps) like the MST (MicroSurgical Technology Company, Nebraska, United States) through the previously done openings nasally and temporally, the Gore-Tex free end will be handled from the Kelman-McPherson forceps which entered through the main wound, and then, the thread of Gore-Tex will be pulled through the openings externally. Then, the other side of the thread from the main wound is passed through the loop haptic of the lens (IOL), and then, this end will be advanced inside the eye from the main wound to be handled again to the tip of MST forceps which entered the globe through the next opening to externalize the other end. Now, we have two free ends through the scleral openings showed outside. The same is done to the opposite side by threading the Gore-Tex suture through the other haptic with two free ends appearing there. What needs to be mentioned is that an extra care should be expressed not to interlock the arms of the suture of the two sides together (Figures 5(f), 5(g), and 5(h)). The next step is to externalize the two free ends of the Gore-Tex from the corneal entrance anteriorly by using the Sinskey hook, carefully adjusting the tension and centralizing the IOL, which followed by doing a 3-1-1 or more knots, which will be easily buried by pushing the knots inside the pockets of each side; no suture is needed to close the pocket. Then, the main wound is closed either by stromal hydration of the cornea or 10-0 nylon suture (Figures 5(i), 5(j), and 5(k)).

## 4. Discussion

Aphakia nowadays has been solved by secondary IOL implantation, which provides better toleration by the patient compared to eyeglasses as management option, with different techniques being invented in the field, including sutured and nonsutured techniques, iris-fixated IOLs, anterior chamber IOLs, and scleral fixated IOLs [2]. Different techniques have been described for scleral fixation of the IOL by novel surgeons over the last four decades, with techniques known by the name of the developers like Malbran, Lewis, Mittelviefhaus, Szurman, Khan, Scharioth, Prenner, Abbey, Yamane, and Hoffman, and these techniques passed through many levels of development with different levels of acceptance [3–13].

In this case series article, we will focus on Hoffman's method for SFIOL implantation. But we will not cover the comparison between different methods of IOL implantation/fixation. However, Hoffman's technique is superior to the other described techniques by sparing the conjunctival tissue with decreased chances to have postoperative suture–related astigmatism, reduced overall operative time, less inflammatory response postoperatively, and faster rehabilitation time [1].

This is the first time the technique for Hoffman's pocket scleral fixation of the IOL is described. A keratome or a supersharp knife is used to create two clear corneal wounds; each one is sized 1 clock hour nasal and temporal of the cornea. Then, a crescent blade or beaver blade is used to create a partial thickness 1 clock hour sized scleral incisions posterior to every corneal incision that was initially created at the limbus area. Then, two partial scleral incisions will be dissected by the beaver blade posteriorly to create the scleral pockets (the length of dissection is 3 mm aiming posteriorly away from the limbus), to create the paracentesis openings which are located anterior to the clear corneal incisions that were created initially in the surgery, and then to use a 27-gauge needle and pass it through the conjunctiva and the scleral pockets at 1 mm posterior to the surgical limbus, entering through the opposite paracentesis corneal incision by a double-armed long needle loaded with a 9.0 polypropylene suture. Then, it should be docked into the 27-gauge needle above or below the subluxated IOL or through the eyelet that has been provided by the manufacturer. Next is to externalize the needle and the suture through the scleral pocket. This step is then repeated again with the other end of the double-armed 9.0 polypropylene suture. After that, we will have the two ends of the fixating suture outside the globe through the scleral pocket openings. Then, we should retrieve these two ends using a Sinskey hook and should tie them together which will result to buried knots [2, 3].

Unfortunately, suture breakage is known with prolene (polypropylene) sutures, but to the best of our knowledge, it was not reported with Gore-Tex material in the ophthalmology field which is advantageous over the prolene suture in the tensile strength and less amount of suture memory and gives lower amount of manipulation intraoperatively giving less inflammatory process postoperatively, although its usage is considered as off label even that it's getting its importance in our ophthalmology field [14]. In addition to this, doing this technique modification with a foldable hydrophobic IOL is adding more advantageous ground in regard to better maneuverability and smaller wound size as compared to the PMMA IOL (rigid polymethyl methacrylate IOL), which usually needs 6 ± 0.5 − mm incision size and globe stability, preventing hypotony and its related complications [1, 2]. Accordingly, in our case series, we used instead of 9.0 polypropylene suture a Gore-Tex (polytetrafluoroethylene) suture with the combination of a foldable IOL RayOne (Rayner company, UK) the hydrophobic type which comes with a dual haptic (loop-shaped) preloaded which to our updated knowledge has not been reported before in the literature to solve IOL implantation issues without capsular support with unique cases including aphakia, complicated cataract surgery, and IOL opacification. That is why we prefer in our practice to use the Gore-Tex sutures to fixate the IOL on the sclera as shown in the provided cases and the surgical technique part above. However, a similar case series has been reported with SFIOL and Gore-Tex sutures and a hydrophilic foldable IOL by Das et al. [3].

## Data Availability

The datasets generated during and/or analyzed during the current study are not publicly available but are available from the corresponding author on a reasonable request.
